# Supplementary material for: Are carbon emissions trading and green financial instruments synergistic? -Comprehensive quantitative research based on content analysis
Source: PLoS One. 2024 Mar 7;19(3):e0298601. doi: 10.1371/journal.pone.0298601 (PMC10919601; doi:10.1371/journal.pone.0298601)
Supplement: S1 Data — (ZIP) [file pone.0298601.s001.zip › Data/地级市Z_F工作报告.zip!/╜¡╬≈/╛┼╜¡╩╨/└┤╫╘╣1⁄2╓┌║┼í╛┬φ┐╦╩2╛▌═°í┐_2003.docx]

各位代表：

我代表市人民政府向大会作政府工作报告，请予审议，并请市政协各位委员和列席会议的同志提出意见。

一、过去五年的回顾及２００３年主要工作

过去五年，在省委、省政府和市委的正确领导下，我们深入贯彻落实党的“十六大”和省第十一次党代会、市第八次党代会精神，努力实践“三个代表”重要思想，弘扬伟大的抗洪精神，迅速完成了灾后重建的艰巨任务，努力实现了恢复发展的既定目标，全面启动了在江西率先崛起的豪迈壮举。这五年，恰逢跨越世纪、千年更迭，在九江发展史上显得很不平凡，有三件大事可以载入史册：一是江湖大堤得以全面整治，增强了我市抗御特大洪水的能力；二是移民建镇工程基本完成，沿江滨湖居民从此有了安居乐业的家园；三是基础设施建设有了突破性进展，城区面貌大为改观，九江跻身中国优秀旅游城市行列。

这五年，综合实力明显增强，显现出在江西率先崛起的良好势头。ＧＤＰ年均增长１２．３％，财政总收入年均增长１１．２％，地方财政一般预算收入年均增长１１．６％，三大产业结构由１９．０：４４．５：３６．５调整为１６．８：４８．０：３５．２。主攻工业取得重大突破，规模以上工业增加值超过５０亿元，累计完成技改项目６９８个，技改投资５５．７５亿元，石油、化工、建材、纺织已成为全省最大的产业集群。国有企业改革取得重大进展，改制面达８７．７％。全市创办１３个工业园，入园企业７００多户，为推进工业化拓展了更大的空间。农业结构调整实现了“两个历史性跨越”，养殖业产值超过种植业产值，经济作物面积超过粮食作物面积。优质棉、水产、蚕桑、药材、花卉苗木等九大优势农产品基地初具规模，龙头企业发展到３９４户。水产品产值年均递增９．９％，完成造林１２１．８万亩，新增封山育林１９０万亩，农垦经济又有长足发展。接待国内外游客年均递增１７．４％，旅游收入年均递增２１．４％。京九农副产品批发市场交易额突破３０亿元，华东装饰材料市场成为全省最大的建材市场，国家级“中国南方粮食交易市场”开局良好。金融机构各项存款余额达２６０．５５亿元，年均增长１５．６％，贷款余额２４２．５２亿元，年均增长８．６％。县域经济在大灾之后奋起直追，主要经济指标的增速在全省的排位大幅前移。

这五年，实施大开放主战略成效显著，开放型经济成为加快发展的主要增长点。建立了“行政服务中心”，开通“经济１１０”，改善投资环境，吸引了日本铃木公司、新加坡金鹰集团、台湾远东集团、香港华孚集团、中国五矿等一批知名企业落户九江。引进外资项目２２２个，实际利用外资６．４亿美元，引进内资项目４０９９个，实际进资２４２．４３亿元，累计出口１．８亿美元。检验检疫、海关、外汇管理等驻市机构和外事、侨务、对台事务、驻外机构在对外开放中发挥了重要作用。九江经济技术开发区投资６．２２亿元完善基础设施，吸引汇源果汁、清源药业等一批项目落户，形成了新的开发态势。共青开发区理顺了管理体制，引进高尔夫球场、航天科技园等重大项目，二次创业势头良好。民营经济不断壮大，民营企业发展到４６４１户，注册资金４５．３亿元，纳税百万元以上的民营企业７６户，销售收入千万元以上的１１９户。

这五年，基础设施建设力度加大，城乡面貌发生了很大变化。累计完成固定资产投资３０３．５亿元，完成重点建设项目４６个。九电三期、柘林电厂扩建、抱子石水电站等工程相继实施，供电量年均增长１３％。完成公路建设１７４７．７公里，鄱阳湖大桥、九景高速、都蔡公路、修武公路相继建成通车。城市基础设施建设累计投资１５亿元，新增市区道路１３２万平方米，新增绿地９０万平方米，新建了长江大道、抗洪大道、十里南大道等１０余条交通主干道，我市被评为全省唯一的国家级“畅通工程二等城市”。白水湖公园、浔阳江广场、新桥头广场等一批园林绿地的建成，营造了良好的休闲环境。全市１２个省级重点镇完成了规划、修编、评审工作。全市电话用户７９．４万户，宽带用户２．２万户。城乡邮路总长１１７７公里，网点１７１处。政府上网工程全面启动，“中国九江网”在全国同类网站中名列前茅。

这五年，社会事业蓬勃发展，人民生活明显改善。实施国家科技计划５７项，取得科技成果９８项，我市被列为江西首批制造业信息化工程示范城市和国家重点联系城市，星火化工厂被确定为国家“火炬计划”有机硅产业基地，科技对经济的贡献率提高到４１％。完成教育投资３８．７９亿元，新增校舍和改造危房１４７万平方米，基本完成企业学校剥离和民师转编工作，农村教育实行“以县为主”的管理体制，双基验收全面达标，民办教育健康发展，九江学院申报成功。卫生体制改革不断深入，防控传染病和重大疾病成效明显，实现了大灾之后无大疫。基本实现广播电视村村通，广电作品获全国性奖项３７个。新闻单位为宣传九江作出了积极贡献。市图书馆建成开馆，九江市志完成总纂，新闻出版事业进一步繁荣，学习型城市创建活动扎实有效。积极实施全民健身计划纲要，竞技体育水平有了提高。社区功能不断完善，荣获“全国社区志愿者先进单位”等六个国家级奖项。城乡居民储蓄存款年均增长１４．５％，城镇居民人均可支配收入年均增长１１．４％。农村税费改革和专项治理为农民减负４７．２％，移民建镇、农民贷款干部担保和扶贫帮困五助工程“三大民心工程”暖民心，有效地改善了城乡人民生活。档案、残联、民族宗教、老龄事业又有新起色，统计、审计、工商、国税、地税、物价、技监、药监等部门在规范市场经济秩序中做出了新贡献。

这五年，可持续发展战略不断推进，社会局面保持稳定。人口出生率控制在１５．２７‰，新增耕地面积４．８万亩，建设了国家级重点生态功能区１个、生态示范区４个、各级自然保护区２９个，水利、防汛抗旱、气象、水文、地震、人防工作在防灾减灾中发挥了重要作用。老建扶贫、库区山区移民投入资金４．５５亿元，改善了贫困地区的生产生活条件，解决了１５．８万贫困人口的温饱问题。“三五”普法基本完成，“四五”普法全面启动，废止涉农收费１０５项，取消行政审批８０８项，办结行政复议申请４６９起，完成了国家城市行政管理综合执法试点工作，成立了安全生产监督机构，建立了长效管理机制。建设社会治安防控体系，严厉打击各种违法犯罪活动和“法轮功”等邪教组织，社会局面祥和、安定。国防教育、民兵预备役工作扎实有效，驻浔解放军、武警、消防、边检在急难险重的关键时刻发挥了重要作用。优抚安置工作卓有成效，获得全国“双拥模范城”两连冠。

本届政府向全市人民所作的七项承诺已基本兑现：１、九江长江干堤工程全面竣工，质量合格率达１００％，抗洪能力提高到防御百年一遇洪水标准，鄱阳湖二期５座重点圩堤的整治工作全面完成。２、４６万沿江滨湖居民完成搬迁，新(扩)建集镇５７个、新村２００个，移民生产、生活条件得到改善。３、农村电网改造基本完成，实现了城乡同网同价。４、实施解困助学工程，减、免学杂费３７８０万元，５０．７８万中小学生受益。５、九威大道、滨江路改造基本完成。６、三水厂(一期)达标超产，东郊垃圾处理场竣工，两湖治理已近尾声，解决了２０万农村人口饮水难问题。７、分流安置下岗职工１０．５３万人，城镇登记失业率低于国家控制标准，两个确保一个低保得到全面落实。

２００３年，我们锁定九江在江西率先崛起的目标，按照市委“一二三四朝前奔”的总要求，克服非典和大旱的严重影响，为实现本届政府提出的五年目标作了紧张而卓有成效的冲刺。实现ＧＤＰ３０６．７７亿元，增长１５．０％，财政总收入２８．６亿元，增长２３．４％，农民人均纯收入２３７３元，增加２５９元。去年的工作主要体现在以下五个方面：

———抗击非典取得阶段性重大胜利。面对突如其来的非典疫情，我们坚定信心、沉着应对、趋利避害、以变应变，迅速启动宣传、防控、救治、联动四大应急机制，拨出专项经费１５００万元，组织严密，防控到位，实现了“力拒非典不进入，确保非典不蔓延”的目标。旅游业排难奋进，积极举办推介活动，“十一”黄金周庐山接待游客创历史新高，星子温泉度假渐成热点，环庐山旅游公路全面动工。部分消费品价格略有回升，农副产品价格全面上扬，消费需求稳中趋旺，粮食、商业、物资、供销、医药、盐业、烟草、石油等行业效益进一步提高。九江国际汽车城、水果交易市场、柴桑大市场、天马建材城建成开业。全年社会消费品零售总额８５．１８亿元，增长１３．８％。

———重点工作有了突破性进展。一是开放型经济增长快。成功举办了“共青论坛”、“海外侨胞江西行(九江)恳谈会”、福州、东京等１２次大型推介活动。引进外资项目９０个，实际利用外资３．２５亿美元，引进内资项目１５３６个，实际引进内资１２６．４５亿元。外贸运量突破１００万吨，集装箱进出口突破１万标箱。二是大项目建设数量多。昌河铃木发动机和整车项目全面动工，赛得利化纤建设加快，亚东水泥二期竣工投产，星火有机硅扩改规模不断扩大，３Ｔ光电、环宇水泥、鑫山水泥、华孚纺织等２６个投资过亿元的项目相继开工上马，投资５０００万元以上的项目３６个。三是固定资产投资增幅高。总量突破百亿元大关，达１１０．４７亿元，增幅为４９．６％，其中民资、外资占投资总额的６０％以上，工业项目占６０％以上。四是城市建设力度大。控制性规划的覆盖率提高到８０％；土地资产储备增加到１１０亿元；九江经济技术开发区的范围调整到１２０平方公里。投资近６亿元加大了中心城区基础设施建设，完成了３０项城建工程，改造了９９条边街小巷，新建改造道路３９万平方米，新增公共绿地２４万平方米，铺设人行道３万多平方米。五是经济自主增长后劲足。财政增长高于ＧＤＰ增长８．４个百分点，规模以上工业增加值增长高于ＧＤＰ增长１３个百分点，经济效益综合指数提高１８．０１个百分点。完成６１家国企改制，落实债务７．６１亿元。

———县域经济发展势头强劲。粮食、棉花、油菜、水产等主要农产品获得较好收成，１家国家级和８家省级农业产业化龙头企业带动农户９．４２万户，建立基地面积２７．２万亩。组建了鄱湖水产股份有限公司。全面启动了“无公害”计划，新申报了５０个省级和３个国家级无公害农产品生产基地，菊三七、云雾茶、山药等地方特色产品开始向标准化迈进，各县(市、区)工业园建设步伐加快，招商引资成效明显，县城建设亮点增多，财政收入过亿元的县(市、区)发展到７个，其中有一个突破２亿元大关，财政增幅跨入全省十快县的有５个县（市、区），农业和农村工作在全省综合评比中荣获第一名。

———社会保障能力进一步增强。发放《再就业优惠证》３．９万份，减免各类税费３９１万元，发放小额贷款１０３０万元，建立了３３个省市级再就业基地，开发社区就业网点２０００多个，全市城镇新增就业５．４万人，其中下岗职工再就业２．２万人，困难群体再就业４７１２人，城镇登记失业率为３．８％。人才市场发育良好，在服务就业中发挥了重要作用。狠抓“两个确保”，发放离退休人员养老金４．６３亿元，发放下岗职工基本生活费及代缴“三金”８５６１万元，市县两级养老金支付率和社会化发放率均达１００％。建立了农村特困群众社会救助制度，１１万人得到了救助，市区居民最低生活保障标准提高到１３０元。劳动保障执法监察工作力度加大，追讨拖欠工资４７４．１万元，有效地维护了农民工的合法权益。

———政府工作坚持透明诚信。大力推行“政务公开”，完善了土地拍卖、工程招标、政府采购等制度，政府１０部门工作向社会承诺兑现，创新了政府工作推进机制。全面完成机构改革任务，４７个政府部门减为３２个，行政编制精减２０％。主动接受人大及其常委会的依法监督，自觉接受人民政协和各民主党派、工商联、人民团体、无党派人士的民主监督，全年共办理人大代表建议、批评和意见１１９件，办理政协提案２６０件。

五年的发展成就，归功于省委、省政府和市委的正确领导，归功于历届市委、市政府打下的工作基础，归功于市人大、市政协、各民主党派、工商联、人民团体的支持和监督，归功于九江人民与时俱进、开拓创新的伟大实践，归功于驻浔单位、驻浔解放军、武警部队的参与和配合，归功于广大海内外朋友的关心、支持和帮助。我代表市人民政府，向所有参与、支持、关心九江建设和发展的同志们、朋友们表示衷心的感谢!

在肯定成绩的同时，我们也必须清醒地看到，当前我市经济社会生活中还存在不少困难和问题：影响经济健康协调发展的体制性和机制性障碍仍然存在，一些深层次的矛盾还没有得到根本解决，直接进入资本市场融资还没有破题；在加快发展中，电力、资金、人才不足的矛盾又凸现出来；工业化、城市化的水平与九江所处的地位不相称；开放型经济的搅动效应、连锁效应和集聚效应没有形成，服务水平有待进一步提高；民营经济所占比重还比较低，县域经济综合实力还不够强，农民增收的难度仍然很大；就业和再就业形势依然严峻，部分群众生活还很困难，社会保障和社会救济压力增大；转变政府职能，提高办事效率还存在不足，廉政建设有待进一步加强。对于这些问题，我们一定高度重视，坚持用改革和发展的办法，努力加以解决。

二、今后五年的奋斗目标和２００４年的主要工作任务

今后五年政府工作的总体要求是：以邓小平理论和“三个代表”重要思想为指导，牢记“两个务必”，深入贯彻党的十六大精神，围绕市第八次党代会提出的九江在江西率先崛起的奋斗目标，牢固树立科学的发展观，坚持“五个统筹”，以大开放为主战略，加快工业化、城市化、农业产业化进程，主攻民营经济，壮大县域经济，增加农民收入，以人为本，关注民生，开创九江全面建设小康社会的新局面。

全市国民经济和社会发展主要预期目标是：到２００８年，实现ＧＤＰ６００亿元，财政收入６０亿元，工业化率４０％，城镇化率４０％，城市居民人均可支配收入年均增长８％，农民人均纯收入年均增长７％，城镇人口登记失业率控制在４％以内，人口自然增长率控制在８‰以内。

通过五年的努力，基本形成经济和社会发展五个新格局：

———基本形成与“长珠闽”对接，融入经济全球化，在更大范围、更广领域、更高层次上扩大开放的格局。积极主动地从体制机制、市场发育、投资服务和基础设施等方面与“长珠闽”三个三角洲对接互动，以市场为主体配置资源，以企业为主体平等竞争，以政府为主体营造环境，使九江成为吸纳投资、集聚财富、汇集人才的全方位开放城市。民营经济、开放型经济在ＧＤＰ和财政收入中所占的比重超过６５％，实现历史性转变。

———基本形成以沿长江沿昌九工业带为主线，以现代制造业为支撑的新型工业化格局。沿江崛起临港工业带，沿昌九崛起工业走廊，园区崛起优势产业群。壮大石油化工、纺织服装、建材冶金、汽车制造、电子电力、食品加工六大支柱产业，形成石化、亚东、金鹰、星火、昌河、九江电厂、３Ｔ光电、７１３厂等一批核心企业。

———基本形成带动全市，服务全省，辐射周边的区域性中心城市格局。建设以中心城区为轴心、一市四区三个卫星镇的大九江，彰显山水个性，打造文化名城，使九江成为长江中下游独具魅力的港口旅游城市。

———基本形成重点突破，特色争先，实力进位，整体推进的县域经济发展格局。按照分类指导，优先发展的原则，引导、支持基础、区位和势头较好的县(市、区)优先发展，力争有一到两个县综合实力跻身全省十强，鼓励、帮助各县(市、区)发挥自身特色，扬优成势，单项争先，在全省乃至全国有影响、有位置。

———基本形成以人为本，关注民生，社会事业协调发展的格局。进一步完善社会保障体系，健全农村特困群众社会救助体系，建立公共突发事件应急机制，壮大教育产业，建设先进文化，统筹人与自然的和谐发展，切实解决与群众生活息息相关的实际问题，确保人民生活水平与经济发展基本同步。

今后五年，重点展开“五十(务实)崛起行动”。一是大力推进１０大基础设施建设。铜九铁路、九武复线、环庐山公路、过境高速、扩改九江港、帽子山核电、西气东输、八里湖城市防洪工程、县城电网改造、病险水库除险保安；二是重点培植１０个税利超亿元的支柱财源企业。九江石化、九江电厂、昌河铃木、亚东水泥、星火化工、赛得利化纤、３Ｔ光电、清华同方７１３厂、金沙湾钢铁、巨石九江工厂；三是精心培育１０个大型市场和物流集团。京九农副产品中心批发市场、华东装饰材料市场、水产批发市场、花卉苗木市场、国际汽车城、天马建材城、南方粮食交易、果品交易、旧货交易、联盛百货；四是倾心实施１０大民心工程。农民贷款干部担保工程，移民搬迁工程，扶贫帮困五助工程，就业和再就业工程，乡村道路改造工程，全民安康工程，农村中小学危房改造工程，环境保护工程，防灾减灾工程，城市弱势群体廉租房工程。五是中心城区建设１０大功能性设施。体育中心、会展中心、青少年活动中心、妇幼保健中心、老年活动中心、文化艺术中心、疾病控制中心、公路客运中心、抗洪纪念馆、城市森林公园。

２００４年，是九江实现在江西率先崛起的关键之年，也是新一届政府的开局之年。全市国民经济和社会发展预期目标是：实现ＧＤＰ３５０亿元，增长１５％；财政总收入３２．９亿元，增长１５％；社会固定资产投资１４０亿元，增长２６．７％；社会消费品零售总额９５．４亿元，增长１２％；实际利用外资３亿美元，增长２０％(同口径)，实际引进内资１５０亿元人民币，增长１８．６％；城镇居民人均可支配收入增长１０％，农民人均纯收入增长８．４％，城镇登记失业率控制在４％以内，人口自然增长率控制在８．０９‰以内。

为了实现良好的开局，今年将从以下八个方面抓好落实：

１、突出农民增收，壮大县域经济，统筹城乡协调发展。

认真贯彻中央１号文件和省委、省政府《实施意见》，切实把“三农”问题放在更加突出的位置，按照统筹城乡经济社会发展的要求，千方百计增加农民收入。

重点帮助粮农促增收。严格执行耕地保护制度，完善征用农民土地的程序和补偿机制，提高补偿标准，妥善安置失地农民。抓住国家实施优质粮食产业工程的机遇，集中力量建设一批国家优质专用粮食基地，扩大沃土工程实施规模，推广优良品种和适用先进技术，促进粮食转化和加工，大力发展养殖业，提高粮食产量、质量和效益，确保粮食安全。加快国有粮食购销企业改革，转变企业经营机制，办好中国南方粮食交易市场，规范搞活粮食流通，将粮食风险基金的４０％直接补贴给粮农，并适当向种粮大户倾斜，提高种粮农民收入。

优化产业结构促增收。坚定不移地推进产业结构调整，抓好棉花、水产、水果、药材、蚕桑、油菜等十大优质农产品基地建设，扶持山区、滨湖地区发展畜牧业、水产养殖和特色林果业。进一步实施龙头带动战略，加速培育“春赐福”、“欢叶”、“芦花”等十大品牌。大力发展农业产业化经营中介组织，重点培植１００户农产品经纪人和营销大户。建立健全农产品质量标准和检验检测体系，加快无公害农产品基地和绿色食品基地建设。加强农村畜牧兽医网点建设，做好禽流感的防控工作。改善农民进城就业环境，加强农民外出务工的就业指导、技能培训，不断提高农村劳动力的就业能力。在税收、信贷、资源使用等方面给予支持，鼓励农民从事二、三产业，拓宽增收渠道。

改善基础设施促增收。加大人畜饮水、乡村道路、农村沼气、农村水电的投入力度，完成９３９公里的县乡道路新建改造，实施３１６国道改造工程，全面完成“平垸行洪、退田还湖、退耕还林、移民建镇”巩固工程，抓好日元贷款防洪工程实施，完成年度病险水库除险加固工程，推进农业综合开发水利骨干工程建设，加快修河、潦河、博阳河、长河及鄱阳湖重点河段的整治和疏浚。完成２．３万村民的饮水工程。加快县城和县级工业园建设，完善重点集镇功能，为解决农村劳动力和人口平稳有序转移提供平台。

壮大县域经济促增收。坚持分类指导、差别政策、非均衡发展，鼓励、引导各县(市、区)发挥优势，创新特色，谋求跨越，在全省争名次、争地位，促进县域经济整体水平的提高。广开门路培植县级财源支柱，增强县级财政统筹能力，充分发挥财政在解决社会公共需要和扶持贫困群众的作用。巩固和发展税费改革成果，加快推进配套改革，加强监督管理，进一步减轻农民负担。继续推进移民建镇、农民贷款干部担保、特困救助“三大民心工程”，加大扶农支农力度，扩大农户小额信贷和农户联保贷款，做好库区山区移民工作，切实解决农民生产生活中的实际困难。

２、优化经济环境，主攻民营经济，提升开放型经济水平。

把市场经济的发育建立在好的环境之上，把增长提速的动力根植于民营之中，以最优的环境、最好的资源、最强的攻势，主攻民营经济，在更高层次上扩大对外开放。

优化环境狠抓三个方面：优化政务环境，转变政府职能，提高办事效率。进一步深化行政审批制度改革，该取消的事项坚决取消，对确需保留的事项，简化程序，规范操作，公开透明，限时办结。充分发挥行政服务中心的作用，敞开民资、外资进入通道。优化法制环境，坚持依法办事，文明执法，完善行政职能运行机制，实行行政资格审查和持证上岗制度，阳光执法。健全严格的行政责任制和行政不作为、行政过错追究、行风评议等制度，切实维护投资者和纳税人的合法权益。优化人文环境，大力营造诚信为本、亲商安商的社会氛围，通过调查研究、现场办公、走访慰问等形式，以情感人、以诚待人，为外商和民营业主提供顺心、顺气、顺手的创业环境。

主攻民营形成四个一批：国企转制成长一批，鼓励民资积极参与国有企业的改制重组、破产兼并、嫁接改造，把国有企业的退出作为壮大民营经济的机遇。对外开放引进一批，坚持内外开放并举，民资外资并重，在引进国内百强民营企业上取得突破。激活民资培育一批，对民营企业在财政、税收、用地、劳动保障等方面给予支持，市县两级成立民营企业担保机构，鼓励行业协会互相担保和联合担保。扶优扶强壮大一批，对骨干民营企业实行领导挂点帮扶，银行授信支持，专家科技联姻，引导其进一步扩大规模，创立品牌，转变管理方式，提升企业档次。认真落实出口退税政策，帮助更多的民营企业获得外贸经营权，发展出口加工型企业。力争今年全市销售收入过千万元的民营企业达１５０户，纳税超百万元的达１００户，外贸出口６０００万美元。

招商引资突出三个重点：突出重点地区，对内瞄准“长珠闽”，境外主攻港澳台地区和日韩等国。扎扎实实办好沿长江工业带项目推介会，集中力量开展招引台商的系列活动，使推介会有目标、创特色、见成效。突出重点平台，办好两个省级开发区，做实县区工业园，进一步完善九江经济技术开发区和共青开发区的基础设施，积极申报国家级开发区和高新技术开发区，做好日本中心工业园建设的前期工作。突出重点项目，做好产业规划、用地规划、项目规划，特别是大项目储备，紧紧咬住有投资意向的大项目不放，实行“领导挂帅、跟踪服务”的包干责任制，确保项目落地生根。

３、狠抓大项目，重点建设沿江工业带，加快工业崛起步伐。

集中力量抓大项目，为培育税利过亿元的大企业打基础。着力抓好２５个投资过亿元的重大项目，大力扶持赛得利化纤、３Ｔ光电、７１３扩改、香炉山钨矿等项目，确保在年内竣工投产；加快金沙湾钢铁、昌河铃木、亚东三期、星火有机硅、汇源乳业等项目的建设进度，力争缩短工期，早日投产；对１００万吨延迟焦化装置、６０万吨甲醇、凤竹纺织等１５个重大项目实行专人跟踪，千方百计把项目引进来。加强调度，通力协作，支持驻市中央、省属企业做大做强。

把建设沿江工业带作为对接“长三角”、实现率先崛起的突破口。加快完成沿江工业带总体开发规划，研究制定沿江开发的产业政策，成立专门机构，集中财力、物力、人力，形成强攻态势。加快沿江地区基础设施建设步伐，高起点改造九江港，改善沿江码头、路网、电网等配套设施，积极促成过境高速公路立项，力争湖口国际集装箱码头开工建设，做好申报出口加工区工作，推进口岸信息平台建设。实施市县联动、工港联动、旅贸联动，大力争取国家和省内规模大、运量大、临港耗水的项目，沿江的六县二区力争都有一至两个大项目进入沿江工业带。

加大力度推进国有企业改制，千方百计提高工业经济运行质量。认真总结前两年国企改革的成功经验，全面推行以产权制度为核心的国企改革工作，在确保国有资产不流失，职工得到妥善安置的前提下，因企制宜，一厂一策，鼓励优势企业兼并嫁接，鼓励民营企业收购重组，推行国企股权多元化改革，确保完成钢铁公司、啤酒厂、制氧机厂等１０户市属国企改革任务，基本完成县(市、区)国企改革任务。

４、完善功能，提升品位，强化管理，打造城市个性。

完善功能。突出完善水、电、路等基础设施，建设１０万吨水厂、八里湖污水处理厂，完成两湖引水置换工程。抓紧城区供电线路的整治，改造输变电设施，确保生产、生活用电。加快环庐山公路建设，打通濂溪大道、青年南路、金凤路等１０条城区干线，启动老龙开河疏浚工程，实施八里湖堤路结合工程，改造南湖支路，开工建设会展中心、体育中心、青少年活动中心和老年活动中心。

提升品位。按照“增绿、增亮、增净、增景”的要求，塑造城市东西南北门户形象。完成长虹大道、滨江中路、十里大道、九瑞大道街景的高标准改造，使之成为四条靓丽的景观大道、形象大道。加快建设抗洪纪念馆、城市森林公园、莲花公园、滨江生态园，为市民提供更多的休闲空间。

强化管理。完善《九江城市总体规划》，编制市域城镇体系规划、历史文化名城保护规划，完成城市管网规划、各个组团的控制性详规、城市园林绿地专项规划和县城总体规划，维护规划的权威性和严肃性，坚决制止各种违法乱建行为。健全市、区、街道、社区相互衔接，合理分工和规范高效的城市管理体系，加大城市管理综合执法力度，从严整治市容市貌和交通秩序，建立城市管理长效机制。从关系市民切身利益的事情做起，着力引导、规范和促进社区建设，增强社区管理和服务功能。

打造个性。重点打好“文化牌”和“山水牌”。充分发挥文化底蕴深厚的优势，体现文化古城的风貌，挖掘历史留下的珍贵遗产，加快琵琶亭、浪井、英租界等一批古遗迹、古建筑的恢复建设。注重引绿、引山、引水进城，营造城在江边，湖在城中，山在窗前的特色环境。

５、培育旅游热点，激活商贸物流，大力发展现代服务业。

实施《九江市旅游发展总体规划》，开展创建“中国最佳旅游城市”活动，加快庐山、柘林湖等旅游景区的经营机制改革，加大旅游集团上市工作力度，引资开发新景点，培育旅游新热点，完善旅游基础设施，努力提升旅游业的综合实力。以昌北机场开通国际航班和九江机场复航为契机，发挥世界地质公园和庐山旅游质量认证两大新优势，在境内外选择热点城市举办大型旅游促销活动，主动融入“长三角”、“珠三角”旅游经济圈和长江、京九旅游经济带，做大境外客源市场，实现全年接待入境游客５．５万人次，外汇收入１７８４万美元，接待国内游客６６０万人次，旅游收入４２．５亿元。

发挥区位优势，激活现代商贸物流业。加快商贸企业产权制度改革，扶持大型商贸连锁企业走向社区。制定现代物流业发展总体规划，着手花卉苗木、水产、旧货等市场的选址立项工作。结合沿江开发，规划物流、港口、仓储基地，采取优惠政策，吸引外商参与大型专业批发市场建设，扶持京九农副产品、华东装饰材料、国际汽车城、天马建材城等市场提升档次、规范管理、做大做强。严厉打击各种破坏市场经济秩序的违法犯罪行为，加强物价监督，保持居民生活必需品的价格稳定。

加快发展金融、证券、保险、中介、咨询、家政、物流配送等现代服务业。继续整治社会信用环境，积极开展创建信用县(市、区)活动，培育信用中介机构，构筑相依、互信、双赢的新型银政、银企关系，加大对民营企业、农村经济和服务业的信贷支持，发展消费信贷，增强防范金融风险能力，降低不良贷款。重视商业保险对社会保障的补充作用，完善多层次保障体系。鼓励发展家政服务业，加强家政服务培训和管理，提高服务质量。进一步规范搞活交通运输市场，加强港口、站场建设，发挥水运优势，确保水运安全，发展高速客运货运，不断壮大物流配送能力。以“全国电子政务试点城市”为契机，建立以电子政务、电子商务、电子社区为基础的综合信息服务体系，构筑“数字九江”的基础框架。

６、高度重视人才，优先发展科教，促进社会事业协调发展。

树立人才资源是第一资源的科学人才观，大力实施人才强市战略。努力建设一支高素质的公务员、专业技术人员、经营管理者和乡土人才队伍。加大实用技术、专业技术、农民工技能培训力度。加强人才市场管理，规范和完善人才录用机制，搞好人才交流服务。

坚持把科教放在优先发展的地位。完善“以县为主”农村九年义务教育管理体制，巩固“双基”成果，完成１６万平方米中小学危房改造，确保乡村教师工资足额发放。着手创建高中园区，加快外国语学校的开工建设。做大做强教育产业，促进九江学院整合提升，加快新的九江师专、九江工程学院组建工作。开放非义务教育市场，鼓励外资和社会力量参与办学。建设教育信息网，构建终身教育体系，营造学习型城市。建设以优势资源为基础的农业、水产科研基地，以骨干企业为主体的研发基地和以开发区、工业园区为依托的高新技术应用基地，大力推进科技成果商品化、产业化。

突出先进文化建设，发展社会各项事业。加强公民思想道德教育，认真贯彻《公民道德建设实施纲要》，开展多种形式的精神文明创建活动。繁荣文化生活，加强文化市场监管，重视文化遗产的保护和利用。进一步扩大广播电视覆盖范围，丰富广播电视节目。深化城镇医药卫生体制改革，重点抓好农村卫生和社区卫生服务，完善农村药品供应网络，继续抓好非典预防工作，建立处理公共卫生突发事件长效机制，提高疫情预警和防治能力。积极开展全民健身运动，重视社区体育发展，努力提高竞技体育水平，培育体育产业。做好出生人口性别比失衡专项整治工作，稳定低生育水平，提高出生人口的素质。切实维护妇女和未成年人、老年人、残疾人的合法权益，为他们创造公平参与社会生活的条件。加强审计监督，坚持依法统计，重视档案管理和综合年鉴编纂工作。加快天气雷达系统和九江水情分析中心建设，搞好人防、地震、水文、地质监测，提高防灾减灾能力。高度重视环境保护，开展“国家环境保护模范城市”创建活动。切实做好外事侨务、民族宗教、新闻出版工作。强化国防教育，搞好国防动员和民兵预备役工作。巩固“双拥”成果，增进军政、军民团结，切实抓好复员转业军人的安置。

７、坚持以民为先，关心群众生活，维护社会稳定。

把关注民生、重视民意、解民所忧作为新的执政观、发展观的重要内容，倾听群众呼声，体察民间疾苦，全面启动“十大民心工程”，多办老百姓企盼的实事。

大力实施就业和再就业工程，让有劳动愿望和能力的人有盼头、有出路。坚持政府引导就业，市场调节就业，个人自主择业，依靠行政和经济手段，完善“一城两园一街”，加大对下岗失业人员的援助力度，建设、规范劳动力市场，加强对大中专毕业生的就业和创业指导，规范各类企业在招工、裁员、工资分配、保险福利等方面的行为，提供公平就业机会，千方百计扩大就业渠道。

完善社会保障体系和社会救助体系，让老弱病残的人有依靠、有归宿。继续巩固“两个确保”的成果，做好“三条保障线”的衔接工作，确保城镇职工基本养老金、下岗职工基本生活费按时足额发放，城镇低保对象应保尽保。进一步实施“五助工程”，扩大贫困群众和农村群众参加医疗保险的比重。建立扶贫济困基金和社会救助体系，切实解决好贫困群众在就医、子女入学、居住等方面的基本生活问题。

筑好“安全、稳定”两道防线，让全社会的人能安心、能放心。强化领导责任制，认真做好信访工作，妥善处理和化解人民内部矛盾，确保不发生影响社会稳定的重大群体事件。坚持“打防结合、以防为主”的方针，加强社会治安综合治理，狠抓政法队伍建设，严厉打击各种刑事犯罪和经济犯罪活动，坚决取缔邪教组织，扫除黄、赌、毒等社会丑恶现象。高度重视人民生命财产安全，建立健全应急救援体系和四级安监网络，强化食品、药品安全，落实安全生产责任制，加大整治力度，消除各类安全隐患，有效预防重大恶性事故发生。

８、坚持执政为民，创新工作机制，切实加强政府自身建设。

全体公务员必须牢固确立以人为本、执政为民的理念，大兴求真务实之风，担当全市人民重托，建设一个“开拓、透明、高效、诚信”的政府。

开拓就是解放思想，转变观念，与时俱进，建设责任的政府。解除阻碍社会发展的一切旧思想、旧观念的禁锢，敢为人先，敢于创新，敢于实践。善于集中民智、借用外智，为己所用，开阔眼界，提升境界。在发展追求上，敢于跨越，争上台阶；在机制体制上，敢于创新，打破传统；在开放开发上，敢冒风险，敢于尝试；在狠抓落实上，雷厉风行，立说立干。

透明就是充分尊重市民的知情权、参与权和监督权，建设廉洁的政府。加强政风建设，加大反腐倡廉力度，把防腐拒变的关口前移，从源头上、机制上预防和治理腐败。围绕市民关心的热点和重点问题，加大政务公开力度，建立和完善重大问题集体决策制度，社会公示和听证制度，提高决策的透明度和公众参与度，政府重大活动情况和结果及时向社会公示，主动接受各阶层的监督。扩大财政财务审计、绩效审计和干部任期经济责任审计结果公布范围。土地出让、工程建设严格实行招投标制度，重大支出一律实行政府采购制度，所有公务员都要权为民用，利为民谋，不搞以权谋私。

高效就是不错位，不越位，不缺位，建设务实的政府。深入基层，贴近群众，调查研究，靠前指挥，做到“有数才有效”。强化政令畅通，加强政务督查，令行禁止，做到“有律才有效”。转变职能，还权于社会，有所为，有所不为，做到“有限才有效”。加强政府行为规范化建设，严格按照规章制度和法定程序办事，力戒随意性，做到“有序才有效”。建立健全承诺监督的推进机制，奖惩分明的制约机制，严格执行服务承诺制、限期办结制、首问责任制、失职追究制，做到“有制才有效”。

诚信就是一诺千金，说到做到，取得市民的认同和信任，建设法制的政府。贯彻实施《行政许可法》，加强行政执法监督，全面推进依法治市进程，政府的一言一行都必须规范在宪法和法律范围内，做到“守法诚信”；对上级党委、政府的方针政策和市委的决策部署，理解到位，执行到位，做到“守责诚信”；凡是既定的目标，部署的工作，确定的事项，实行责任到人，事事追踪，逐项完成，做到“守诺诚信”。主动接受人大的法律监督、政协的民主监督、社会舆论监督和群众监督，认真办理人大代表建议、政协委员提案，充分发挥工会、共青团、妇联等群团组织的桥梁纽带作用。

各位代表，市政府工作报告经大会审议通过后，就意味着市政府向全市人民作出了庄严承诺，唯有做好九江发展的大事，市民关注的实事，外商提出的急事，才是本份。我真诚希望社会各界关心、支持政府的工作，大家一起共同把九江的事情办好。未来五年，是我们发挥后发优势的“机遇期”，也是释放巨大能量的“崛起期”。让我们紧密团结在以胡锦涛同志为总书记的党中央周围，高举邓小平理论伟大旗帜，全面贯彻“三个代表”重要思想，在省委、省政府和市委的正确领导下，弘扬伟大的抗洪精神，与时俱进，开拓进取，为实现九江在江西率先崛起而努力奋斗!
